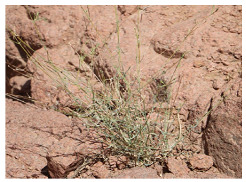# Oxidative Stress Responses of Some Endemic Plants to High Altitudes by Intensifying Antioxidants and Secondary Metabolites Content

**DOI:** 10.3390/plants9070869

**Published:** 2020-07-09

**Authors:** Ahmed M. Hashim, Basmah M. Alharbi, Awatif M. Abdulmajeed, Amr Elkelish, Wael N. Hozzein, Heba M. Hassan

**Affiliations:** 1 Botany Department, Faculty of Science, Ain Shams University, Cairo 11865, Egypt; hashim-a-m@sci.asu.edu.eg (A.M.H.);; 2 Biology Department, Faculty of Science, Tabuk University, Tabuk 71421, Saudi Arabia; b.alharbi@ut.edu.sa; 3 Biology Department, Faculty of Science, Tabuk University, Umluj 41912, Saudi Arabia; 4 Botany Department, Faculty of Science, Suez Canal University, Ismailia 41522, Egypt; amr.elkelish@science.suez.edu.eg; 5 Bioproducts Research Chair, Zoology Department, College of Science, King Saud University, Riyadh 11451, Saudi Arabia; 6 Botany and Microbiology Department, Faculty of Science, Beni-Suef University, Beni-Suef 62511, Egypt

**Keywords:** altitudinal variation, antioxidant activity, bioactive compounds, endemic species, oxidative damage

## Abstract

Most endemic plant species have limited altitudinal ranges. At higher altitudes, they are subjected to various environmental stresses. However, these plants use unique defense mechanisms at high altitudes as a convenient survival strategy. The changes in antioxidant defense system and accumulation of different secondary metabolites (SMs) were investigated as depending on altitude in five endemic endangered species (*Nepeta septemcrenata*, *Origanum syriacum* subsp. *Sinaicum*, *Phlomis aurea*, *Rosa arabica*, and *Silene schimperiana*) naturally growing in Saint Katherine protectorate (SKP). Leaves were collected from different sites between 1600 and 2200 m above sea level to assess the biochemical and physiological variations in response to high altitudes. At higher altitudes, the soil pH and micronutrient soil content decreased, which can be attributed to lower mineralization processes at lower pH. Total phenols, ascorbic acid, proline, flavonoids, and tannins increased in response to different altitudes. SMs progressively increased in the studied species, associated with a significant decrease in the levels of antioxidant enzyme activity. *R. arabica*, as the most threatened plant, showed the maximum response compared with other species. There was an increase in photosynthetic pigments, which was attained via the increase in chlorophyll a, chlorophyll b, and carotenoid contents. There was a significant increase in total soluble sugars and total soluble protein content in response to different altitudes. SDS-PAGE of leaf proteins showed alteration in the protein profile between different species and the same species grown at a different altitude. These five species can adapt to high-altitude habitats by various physiological mechanisms, which can provide a theoretical basis for the future conservation of these endangered endemic species in SKP.

## 1. Introduction

Mountains are characterized by unique biodiversity. Most plant species at high altitudes are isolated and have a small number of niche habitats in comparison to lowland plants [1]. Consequently, the plant populations of the mountains have a higher percentage of endemism than lowlands plants [2,3]. Endemic species are most frequently endangered at high altitudes because of their limited altitudinal distribution ranges [4,5]. Climate limitations primarily affect the high-altitude ecosystems and most plants flourish only near their climatic limits [6,7].

The region above 2800 m above sea level (a. s. l.) in Saint Katherine protectorate (SKP), Sinai Peninsula, is a notable area, not only for its natural landscapes, but also its diversity of medicinal plants of national and global interest [8,9]. SKP contains a wide range of habitats that are a consequence of varying climatic conditions, altitudes, and topography [10]. Being one of the most floristically diverse locations in the Middle East, SKP accounts for about 44% of Egypt’s endemic plant species [11,12]. Sinai is home to approximately 1285 species, of which approximately 800 species are recorded in its southern region [13]. Previous studies have identified several endemic species among Egyptian flora [14]. Approximately 60 endemic plant species have been tabulated in Egypt, including 31 species on the Sinai Peninsula (i.e., 51.6% of Egyptian endemism) [15]. Twenty-four of these are endemic to South Sinai, and are known for their medicinal properties and use in traditional therapy and remedies [16]. 

Due to the high altitude of SKP, harsh and complex climatic conditions reduce the distribution and diversity of plants [8,17]. These hard conditions include reduced O_2_ and CO_2_, strong winds, high solar irradiance, shallow rocky soils, low temperatures, and low water and nutrient contents [18]. Although these stress conditions could retard plant growth, many species have survived and developed different adaptive mechanisms under these circumstances [19]. 

Accumulation of secondary metabolites (SMs) has been reported to play a vital role in tolerance against different environmental biotic and abiotic stresses [20,21,22,23,24]. Actually, these SMs help in adaptation of stressed plants to different environmental conditions; for example, accumulation of flavonoids and phenols as antioxidants; increase in proline and soluble protein contents against drought; and increased carotenoids compared to chlorophyll to reduce high light intensity damage, through upregulation of the xanthophyll-cycle pigment pool [25,26]. Several factors, such as age, season, and nutrition status, are determinants affecting the quantity of secondary metabolites in plants [27,28]. Similarly, environmental factors significantly cause quantitative and qualitative variations in the production of secondary compounds among plants and population groups [29]; these factors include altitude, high/low temperature, drought, and light conditions [30,31]. 

Combinations of high-altitude environmental stress lead to increased reactive oxygen species (ROS) production, which increases the risk of oxidative damage [32,33,34]. Many enzymes of antioxidative activity, such as ascorbate peroxidase, catalase, superoxide dismutase, and glutathione reductase, have the capacity to scavenge different ROS, including superoxide, hydroxyl radicals, and singlet oxygen [35,36,37]. It was previously confirmed that high mountain plants species are acclimated to high irradiance and chilling stress [38]. High antioxidant and carotenoid levels have been positively associated with the altitude of alpine plants. However, in high mountain plants, the ability of the antioxidant system and the xanthophyll cycle differed considerably [39]. 

In spite of the ecological significance of these endemic species and many of their promising characteristics, their ecophysiology has not been closely studied, particularly at the SKP high altitudes [40,41]. Consequently, knowing how such adaptations influence plant growth is relevant for researchers who wish to predict the response of endemic species in a future scenario of climate change. In the present work, five endemic and endangered species samples were collected from three different sites with varying altitudes in SKP. This study investigated the SMs and antioxidant defense capacity in plant samples from different altitudes, to test the hypothesis that plants at high altitudes have greater defense capacity than those at lower altitudes. In addition, the present work intended to investigate the eco-physiological responses of some endemic species as a result of their exposure to the stresses prevailing at high altitudes of the SKP mountains.

## 2. Results

### 2.1. Soil Analysis

Analysis of soil samples at different heights of SKP mountains showed that soil samples were slightly alkaline, and the pH value decreased with an increase in altitude (Table 1), showing a significant decrease at the highest altitude (2000–2200 m a.s.l.). Elemental analysis of the soil samples (Table 1) showed a reduction in some macronutrients (Na^+^, SO_4_^−^, and Cl^−^), while HCO_3_^−^, Mg^+2^, and Ca^++^ were higher at the middle altitude. Finally, K^+^ increased with the increase in altitude.

### 2.2. Phytochemical Assay

#### 2.2.1. Change in the Photosynthetic Pigments

**Figure 1:**
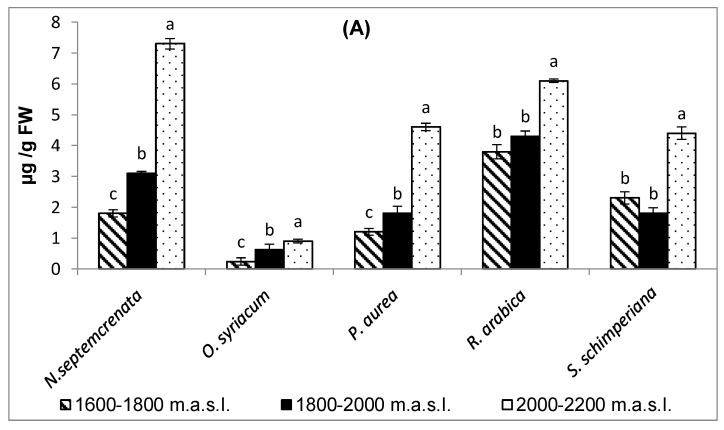

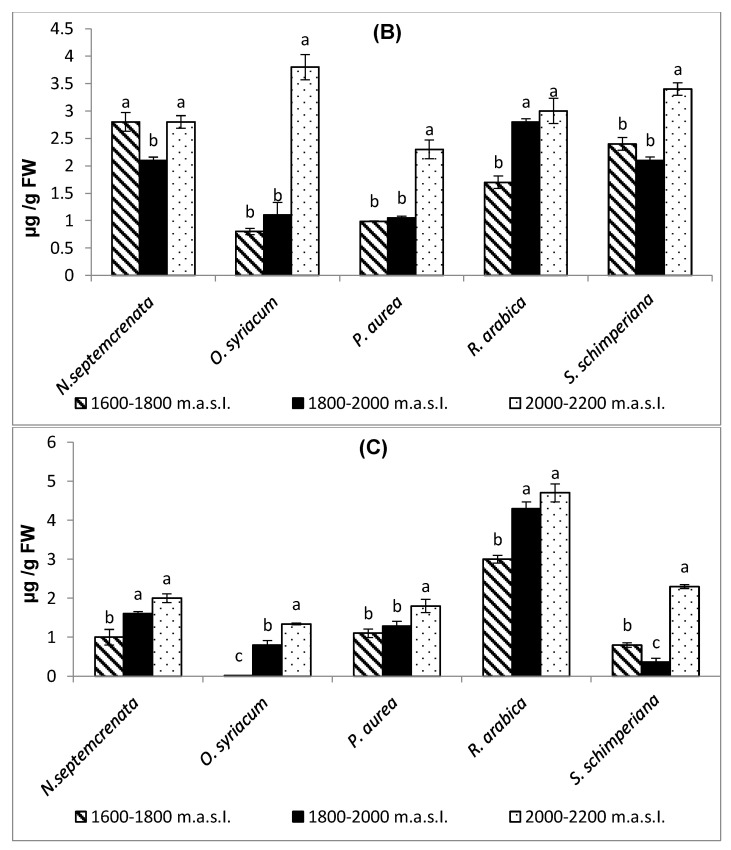
Effect of altitude on the photosynthetic pigments chlorophyll a (**A**), chlorophyll b (**B**), and carotenoids (**C**). Each value is the mean of three replicates ± SE. Bars with different letters are significantly different at *p* ≤ 0.05.

The photosynthetic pigment is shown in Figure 1. Chlorophyll a was considerably increased by moving to a higher altitude (Figure 1A). In addition, chlorophyll b was significantly raised with higher altitude, with the exception of *N. septemcrenata*, which exhibited lower content of chlorophyll b at 1800–2000 m a.s.l. Finally, as altitude increased, carotenoids showed a gradual rise in content, similar to that of chlorophyll a, in the five plant species, except for *S. schimperiana* (Figure 1B). 

#### 2.2.2. Changes in the Content of Major Secondary Metabolites

**Figure 2:**
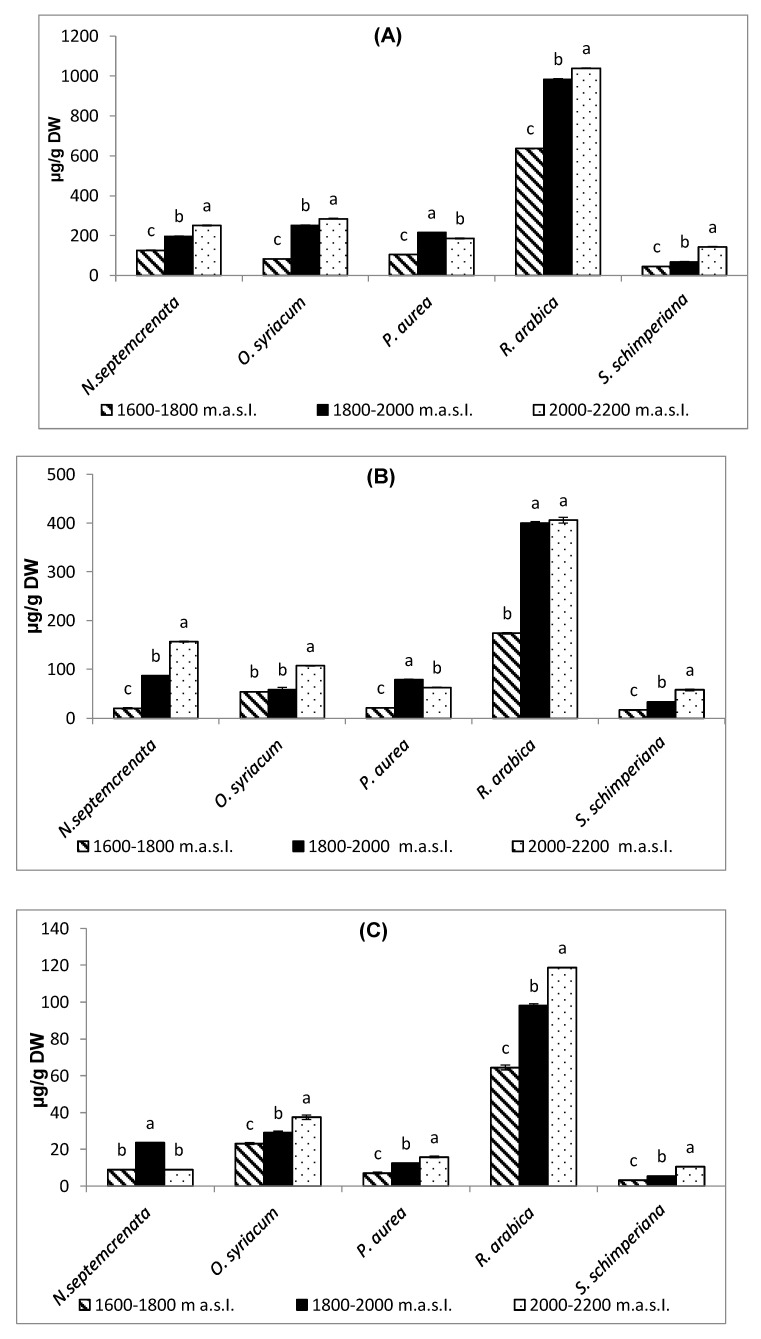
Effect of altitude on the contents of total phenols (**A**), flavonoids (**B**), and tannins (**C**). Each value is the mean of three replicates ± SE. Bars with different letters are significantly different at *p* ≤ 0.05.

It is clear from Figure 2 that, with few exceptions, as the altitude in the SKP mountains increased from 1600 to 2200 m a.s.l. there was a significant increase in the content of total phenols, flavonoids, and tannins of the five plant species under investigation. Within the five plant species, *Rosa arabica* recorded the highest values of total phenols, flavonoids, and tannins at the highest altitude (2000–2200 m a.s.l.).

#### 2.2.3. Changes in the Content of Total Soluble Sugars 

**Figure 3:**
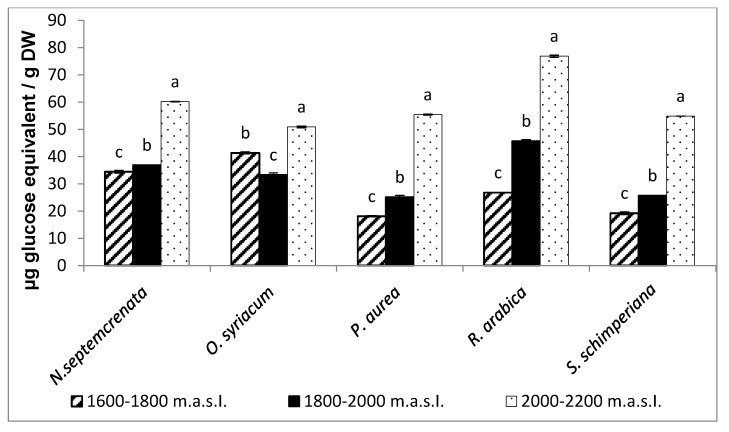
Effect of altitude on the total soluble sugars. Each value is the mean of three replicates ± SE. Bars with different letters are significantly different at *p* ≤ 0.05.

In all studied species, total soluble sugar content increased in all samples from higher altitudes compared to those from lower altitudes. These increases reached 60 and 76.85 µg/g DW in *N. septemcrenata* and *R. arabica*, respectively, grown at 2000–2200 m a.s.l. (Figure 3). 

### 2.3. Biochemical Analysis

#### 2.3.1. Changes in the Content of Total Soluble Proteins

**Figure 4:**
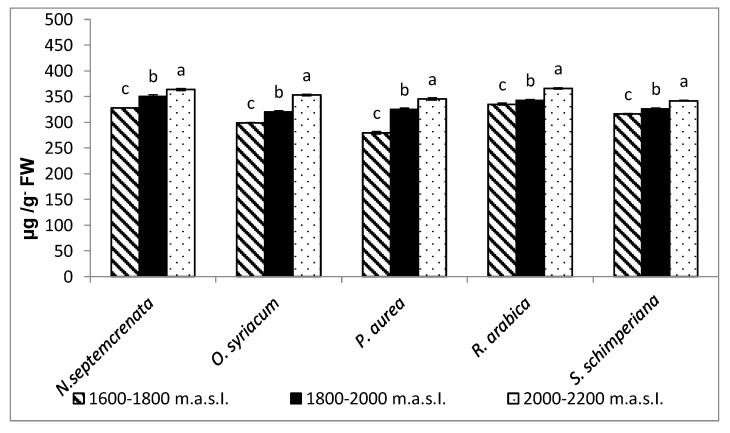
Effect of altitude on the total soluble proteins. Each value is the mean of three replicates ± SE. Bars with different letters are significantly different at *p* ≤ 0.05.

Total soluble proteins in response to high altitudes showed high concentrations in all sampled plants at the three studied sites. *N. septtemcrenata* and *R. arabica* attained the highest values of total soluble proteins (363.6 and 365.93 µg/g FW), respectively (Figure 4).

#### 2.3.2. Determination of Protein Banding Pattern 

**Figure 5:**
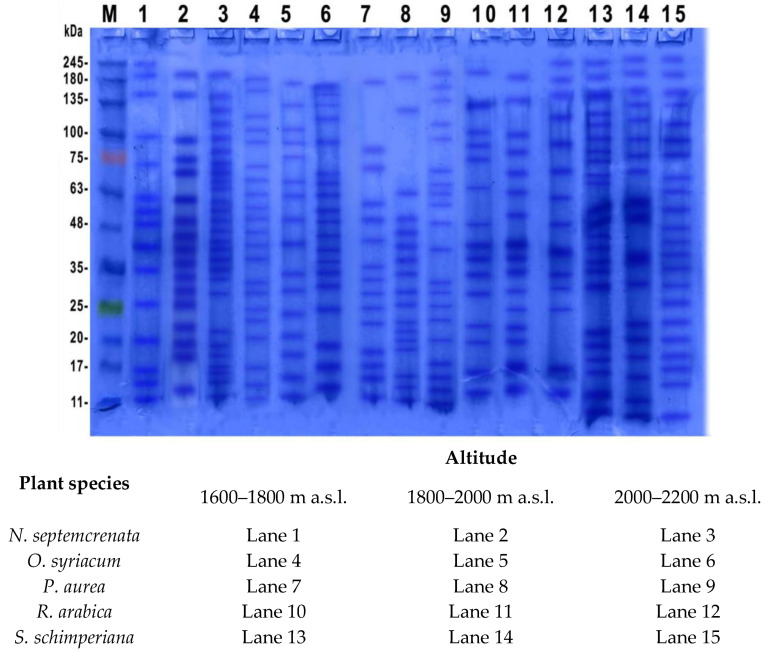
Change in protein banding pattern of different five plant species grown at different altitudes of SKP mountains.

Plate 1 shows the change in protein banding patterns of the five species under investigation in response to a change in altitude of the SKP mountains from 1600 to 2200 m a.s.l. SDS-PAGE (Figure 5) revealed the presence of common bands in the leaves of different plant species grown at the same altitude of the SKP mountains. Common bands detected in plants grown at the relatively low altitude (1600–1800 m a.s.l.) were 70.63, 48, 40, 35, 17, 15, and 11 KDa. Nine protein bands (135, 100, 75, 48, 35, 25, 20, 15, and 11 KDa) were detected in protein extracts of plants grown at 1800–2000 m a.s.l. and nine protein bands (180, 135, 100, 48, 35, 24, 18, 15, and 11 KDa) in plant species grown at the highest altitude (2000–2200 m a.s.l.).

#### 2.3.3. Changes in the Proline Content 

**Figure 6:**
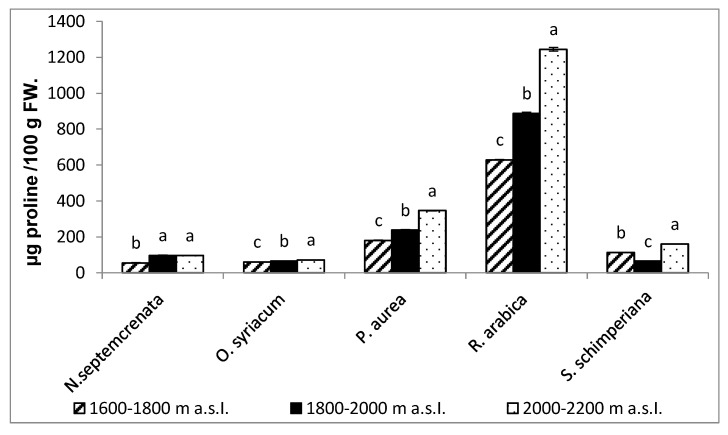
Change in proline contents for five plant species grown at different altitudes of SKP mountains. Each value is the mean of three replicates ± SE. Bars with different letters are significantly different at *p* ≤ 0.05.

Data shown in Figure 6 indicate that, among all species and at different altitude levels, *Rosa arabica* recorded the highest value of proline content (1244.7 µg/100 g FW), especially at the highest altitude (2000–2200 m a.s.l.).

#### 2.3.4. Changes in Total Antioxidant Capacity and Malondialdehyde (MDA) Contents 

**Figure 7:**
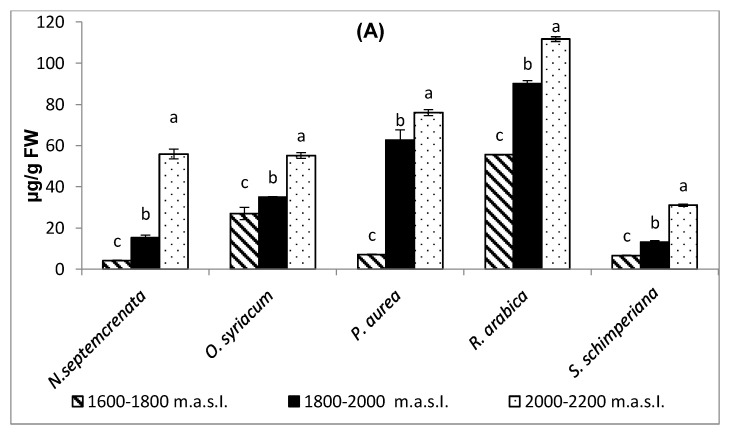

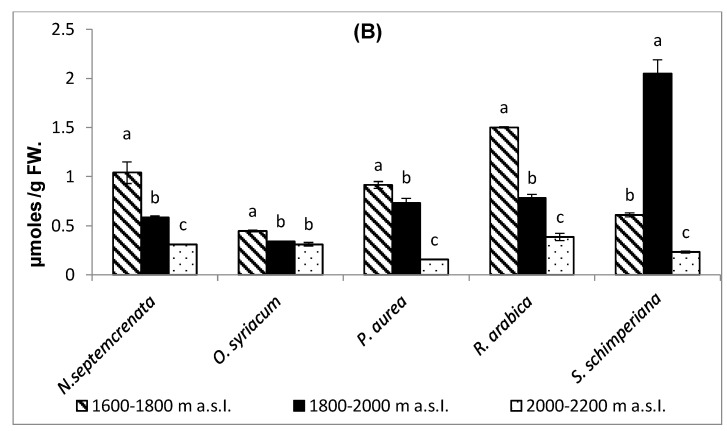
Effect of altitude on the total antioxidant capacity (**A**) and the content of malondialdehyde (**B**). Each value is the mean of three replicates ± SE. Bars with different letters are significantly different at *p* ≤ 0.05.

The increase in altitude induced a significant increase in total antioxidant capacity of the five investigated species, with the highest value in *R. arabica* (111.7 µg/g FW) at the highest altitude (2000–2200 m a.s.l.); Figure 7A. Concomitant with the increase in total antioxidant capacity, the content of MDA was mostly significantly decreased. MDA was used as an indicator for lipid peroxidation and hence oxidative stress; the increase was more pronounced in *S. schimperiana* (located at altitude 1800–2000 m a.s.l.) and *R. arabica* (located at first altitude 1600–1800 m a.s.l.) with values of 2.05 and 1.5 µmoles/g FW, respectively, as shown in Figure 7B.

#### 2.3.5. Change in Activity Level of Some Antioxidant Enzymes 

**Figure 8:**
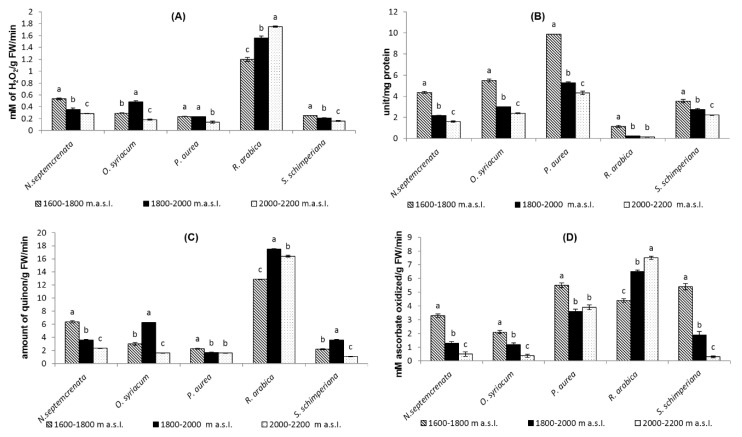
Effect of altitude on the activity of some antioxidant enzymes, catalase (**A**), superoxide dismutase (**B**), ascorbic acid peroxidase (**C**), and polyphenol peroxidase (**D**). Each value is the mean of three replicates ± SE. Bars with different letters are significantly different at *p* ≤ 0.05.

The present findings reveal that catalase (CAT), superoxide dismutase (SOD), polyphenol peroxidase (POD), and ascorbate peroxidase (APX) activity level decreased with altitude increase in all species, with the exceptions of *O. syriacum* (Figure 8A,C) and *R. arabica* (Figure 8A–D). CAT, POD, and APX activities of *R. arabica* increased significantly with increase in altitude and recorded their highest values (1.75 mM/g FW/min, 16.4 amount of quinon/g FW/min, and 16.5 mM ascorbate oxidized/g FW/min, respectively), at the highest altitude (2000–2200 m a.s.l.), whereas SOD activity increased to the maximum value (9.89 unit/mg protein) in *P. aurea* at the lowest altitude in our study (1600–1800 m a.s.l.).

#### 2.3.6. Change in Ascorbic Acid Contents 

**Figure 9:**
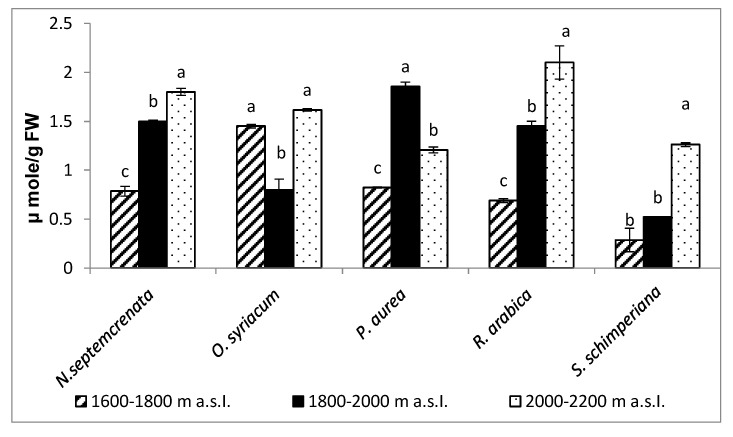
Change in contents of ascorbic acid of different five plant species grown at different altitudes of SKP mountains. Each value is the mean of three replicates ± SE. Bars with different letters are significantly different at *p* ≤ 0.05.

Data represented in Figure 9 indicate that there was a significant increase in the content of ascorbic acid in most plant species with increase in altitude, with the exceptions of *O. syraicum* and *P. aurea*. The highest values (1.8 and 2.1 µmole/g FW) were recorded in *N. septemcrenata* and *Rosa arabica*, respectively, at the highest altitude (2000–2200 m a.s.l.).

## 3. Discussion

In mountainous environments, the altitudinal gradient is associated with a wide variation in environmental conditions, which affect plant distribution and population structure [42]. In high mountain regions, plants are challenged by unfavorable or even adverse abiotic environmental conditions that affect growth dynamics and threaten their existence. This is particularly the case for endemic endangered species [6]. With increasing altitude in the SKP mountains, there is a marked decrease in the distribution and intensity of the growth of plant species [43]. This may be due to the predominance of unfavorable climatic conditions at the highest region of the SKP mountains. The results obtained in the present work (Table 1) indicated that, with the increase in altitude in the SKP mountains, there is a decrease in soil pH and deficiency in macronutrients, which may be due to lower mineralization prevailing at low pH [44]. Other adverse abiotic factors of SKP as a highly elevated region include low temperature and deficiencies of oxygen [45,46], water precipitation, light intensity, and UV radiation [47,48].

Generally, in natural systems, a complex interplay between abiotic stressors and plant growth has resulted in several physiological traits by adaptation, acclimation, and speciation, which may differ between different plant species [49]. The photosynthetic process responds to the environment and maintains homeostasis by displaying several forms of adaptation [50]. Some secondary metabolites actively participate in this issue, particularly in the inhibition of chlorophyll photo-oxidation and accumulation of free radicals [51]. Moreover, as a derivative of photosynthetic products furnishes the secondary metabolic pathway with certain intermediates, this enhances photosynthetic performance via the positive feedback mechanism. The results obtained in the present work showed that with the increase in altitude there was a significant increase in the content of total soluble sugars (Figure 2) and total soluble proteins (Figure 4) in the plant species under investigation, with the highest content of total soluble sugars recorded in *Rosa Arabica* grown at 2000–2200 m a.s.l. In accordance with these results, Castrillo [52] found that higher altitudes triggered the accumulation of total soluble sugars in *Espeletia schultzii*. These data may reflect a higher photosynthetic performance of plants grown at the highest altitude. The data could be explained based on the increase in the concentration of HCO_3_^−^ and Mg^+2^ in the soil of the highest altitude region (Table 1). Magnesium, as a component of the chlorophyll structure and a cofactor of some enzymes of the photosynthetic process, may enhance the photosynthetic process [53,54]. Moreover, there was a significant increase in the content of chlorophyll a, chlorophyll b, and carotenoids (Figure 1). Carotenoids, in addition to being active as light-harvesting pigments [55,56], act as a scavenger of singlet oxygen species and quench the triplet state of chlorophyll molecules [57].

Under abiotic stress, cellular homeostasis is disrupted, leading to the production of free radicals, which in turn can result in oxidative damage [58,59]. Moreover, in the high mountain region of SKP, among the more aggressive stressors are high light intensity and UV-B radiation [60]. Under these conditions, it is possible that electrons released from excited chlorophylls are transferred from photosystem I of the photosynthetic process to O_2_ to form superoxide radicals, which initiate a chain of free radical liberation [61]. Hydrogen peroxide at low concentrations acts as a signaling molecule to induce the defense responses of plants under stresses but, at high levels, they may cause a substantial disturbance in the metabolism through damage of lipids of membranes and nucleic acids, conformational changes of enzymic proteins, destruction of thiol-containing compounds, etc. [62]. Soluble sugars were reported to be involved in defense mechanisms against stress via their efficiency in balancing ROS [63]. In addition, soluble sugars were reported to play a role in cold acclimation of plants [64]. SDS-PAGE of the protein extract of leaves of the five plant species under investigation is illustrated in Figure 5. The results refer to some differences in the pattern of the protein bands between different plant species and in the same species grown at different altitudes.

Virtually all organisms respond to environmental stress with the synthesis of a specific type of proteins [65]. However, in view of plant growth in SKP under a multitude of stressors, it would seem reasonable to detect the expression of a wide range of proteins. SDS-PAGE (Figure 5) reveals the presence of characteristic bands in the leaves of different plant species grown at the same altitude of the SKP mountains. The protein band with a molecular weight of 35 KDa, which was detected in various plant species irrespective of SKP altitude, is identified as one of the jasmonate-induced proteins that plays a crucial role in the defense against biotic and abiotic stresses [66]. Moreover, the protein with a molecular weight of 48 KDa, which has been identified to function as a proteinase inhibitors [67], was recorded in the five species grown at the three levels of the SKP mountains. In addition, the low molecular weight proteins having an antifungal activity (11 KDa) were detected in all plant species irrespective of the level of altitude of the SKP mountains. It must be stressed that the interpretations of these bands are speculative and require further in-depth investigation using advanced mass spectroscopic identification. Proline accumulation was reported to play a role in adaptation to stress or a consequence of stress [68]. It plays a role in redox homeostasis against ROS; it acts as an antioxidant [69]. In addition, proline acts as a molecular chaperone, capable of defending protein integrity from ROS [70].

A consequence of the abiotic stress prevailing in the SKP mountains, particularly at the highest altitude, is the qualitative and quantitative increase in a variety of SMs as flavonoids, phenolic compounds, alkaloids, carotenoids, steroids, tannins, and terpenoids [71,72]. In the present work, with the increase in altitude of the SKP mountains from 1600 to 2200 m a.s.l., there was a significant increase in the content of proline, total phenols, flavonoids, and tannins (Figure 6 and Figure 3). In addition, phenolic antioxidants inhibit lipid peroxidation by trapping the free radicals, stabilize membranes by decreasing membrane fluidity, hinder the diffusion of free radicals, and restrict peroxidative reactions [73]. Through these interactions, the phenolic and flavonoid compounds increased the adaptation to abiotic oxidative stress. Tannins, on the other hand, have a crucial role against oxidative stress, particularly at high light intensity [74]. Tannins minimize oxidative damage via scavenging free radicals [75]. Tannins also bind to membranes, and the tannin-phospholipid complex may serve to moderate membrane morphology and permeability [76]. It is worth mentioning here that the plants with higher exposure to UV-B triggered phenylalanine ammonia-lyase. Phenylalanine ammonia-lyase is the key enzyme in the biosynthesis of the most important secondary metabolites [77], although these metabolites play a crucial role in plant adaptation to their adverse environmental conditions [78,79]. In addition to the adaptive function of SMs under adverse environments, it plays a crucial role in the maintenance of the stability of the primary metabolism, protecting it from disturbance induced by accumulation of specific metabolites via its conversion into secondary metabolites [80].

Plants successfully grown under oxidative stress actively initiate antioxidant systems, playing a role in their adaptation. Antioxidant defense systems can combat oxidative stress via scavenging of free radicals [81]. The results obtained (Figure 7, Figure 8 and Figure 9) indicated that plant species grown at the relatively lower altitude (1600–1800 m a.s.l.), at which plants grow under relatively mild stress conditions, depend on scavenging free radicals on both the antioxidant enzymes and the antioxidant compounds [30,82]. A reverse pattern of change was observed in the activity level of antioxidant enzymes (catalase, superoxide dismutase, ascorbic acid peroxidase, and polyphenol peroxidase) in all species, with the exception of *R. arabica*, in which the increase in altitude mostly coincided with a corresponding increase in the activity level of antioxidant enzymes (Figure 8).

The modes of action of different antioxidants in the relief of oxidative stress vary [20,33,83]. Thus, plants that grows under stress conditions have many antioxidant molecules that represent the second line of defense against ROS, including ascorbic acid, carotenoids, glutathione, and phenolic compounds [84]. Ascorbic acid is considered the most potent antioxidant in plant tissues due to its capability of donating free electrons in many non-enzymatic and enzymatic reactions. Furthermore, ascorbic acid can scavenge O_2_^•−^ and OH^•^ directly with greater ability to regenerate the oxidized carotenoids and, consequently, provide great protection to the cell membrane and minimize the oxidative damage synergically with other antioxidants [82].

Differences in environmental conditions (such as illumination, temperature, soil characteristics, and altitude) strongly contribute to the antioxidant activity and the amount of active ingredients in endemic medicinal plants [85]. In this study, many significant variations were detected in the antioxidant activity and chemical composition of five endemic targeted species collected from three different altitudes. It is clear that *R. arabica* depends mainly on increasing the activity of its antioxidant enzymes to adapt to high altitude, while the other investigated species tend to rely on antioxidant compounds as an adaptive response against high altitude to survive. Finally, the great variation in antioxidant activity and SM quantity and quality in these plants will possibly lead to considerable differences in their efficacy as herbal medicines [85].

## 4. Materials and Methods 

### 4.1. Study Area

**Figure 10:**
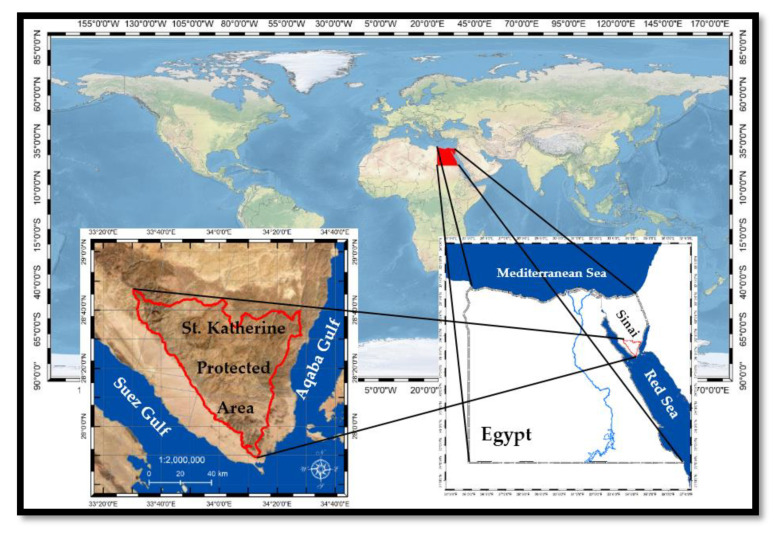
Study area map for Saint Katherine protected area, Sinai, Egypt (Using Landsat8 OLI 2019, Path/Row 178/39).

The current study was conducted in the south part of Sinai, specifically, in the mountainous region of SKP (Figure 10), which was declared a protectorate area by the Egyptian Environmental Affairs Agency (EEAA) in 1996. SKP is Egypt’s fourth largest protectorate and is located between 28°30′ to 28°35′ N and 33°55′ to 34°30′ E. Its plateau altitude ranges between 1300 and more than 2600 m above sea level [43,86]. The area encompasses approximately 180 km^2^ and is characterized by the presence of the highest rugged mountains in Egypt, namely Gebel Catherine (2624 m) and Gebel Mousa (2285 m), and the adjoining peaks [42]. This mountainous arid habitat supports an astounding biodiversity and a high share of rare and endemic plants because of its unique geological, morphological, and climatic aspects [43,87].

The plant materials were collected and field measurements carried out in April 2019 in mountainous habitats of SKP. The study area has been classified as one of the hyper-arid zones of the peninsula. SKP is the coolest area in Sinai owing to its high altitude [8,88]. The mean temperatures range from 5.4 to 25.2 °C, with the lowest temperature in January and February and the highest temperature in July and August [89]. Ayyad et al. [13] stated that high mountains in the SKP receive higher amounts of precipitation (100 mm/year) as rain and snow. The low altitude sites are climatically characterized by very dry summers with 5–30 mm precipitation per year. On the other hand, the high altitude district of South Sinai receives 35–50 mm of precipitation per year [90,91].

### 4.2. Target Species

Five endemic species were collected from three different sites with varying altitudes in the SKP mountains. For each species, five individual plants were taken and, for phytochemical and biochemical measurements, three replicates were taken from each. Details of the target species, including their scientific names with their families, altitude, and field photo are provided in Table 2. Samples from the shoot systems of the five plant species under investigation (namely, *Nepeta septemcrenata* Benth., *Origanum syriacum* subsp. *Sinaicum* (Boiss.) Greater and Burdet., *Phlomis aurea* Decene., *Rosa arabica* Crep., and *Silene schimperiana* Boiss.) were collected from three different altitudes of the SKP mountains (1600–1800, 1800–2000, and 2000–2200 m a.s.l.) and either kept frozen in a deep freezer (−20 °C) for extraction and estimation of enzyme, proline, photosynthetic pigments, total antioxidant capacity, ascorbic acid, malondialdehyde, and protein electrophoresis, or air-dried for extraction of carbohydrate and phenolic compounds. The identification of the five plant species was confirmed with the help of the Herbarium Section, Botany Department, Faculty of Science, Ain Shams University.

### 4.3. Soil Analysis

For each studied altitude, three soil samples were collected from profiles of 0–50 cm depth. Then, air-dried and thoroughly mixed together to form one composite sample. Textures were determined by sieving method to separate gravels, coarse sand, fine sand, silt, and clay. Determination of electric conductivity and pH was determined in soil–water (1:5) extracts using the potentiometric method [92,93]. Calcium and magnesium were determined volumetrically by the versene titration method described by Johnson and Ulrich [94]. Sodium and potassium were determined by flame photometry according to Shapiro and Brannock [95]. Estimation of chlorides was carried out by titration methods using 0.005N Silver Nitrate [93,96]. Sulphates were determined according to Bardsley and Lancaster [96].

### 4.4. Phytochemical Assay

#### 4.4.1. Extraction and Estimation of Photosynthetic Pigments

The photosynthetic pigments including (Chl a, Chl b, and carotenoids) were extracted in 80% acetone and then estimated colorimetrically using Spectronic 601, Milton Roy company, USA according to the method described by Metzner et al. [97].

#### 4.4.2. Extraction and Estimation of Total Soluble Sugars 

Sugars were extracted according to Homme et al. [98], the air-dried tissue was boiled in 80% (*v/v*) ethanol, the filtrated extract was oven-dried at 60 °C, and then dissolved in water and was made to a known volume with water. The total soluble sugars contents were estimated with the anthrone reagent following the method of Loewus [99]. Finally, the concentration of soluble sugar was determined from the standard curve of glucose and calculated as µg glucose equivalent/g DW.

#### 4.4.3. Extraction and Estimation of Total Phenolic Content 

The total phenolic content was extracted and estimated by following the method adopted by Malik and Singh [100]. After extraction with ethanol (80%, *v/v*), the total phenolic content was estimated by Folin and Ciocalteau’s reagent and the optical density of the reaction mixture was read at 750 nm. The concentrations were calculated from a standard curve of pyrogallol as gallic acid equivalents/g.

#### 4.4.4. Extraction and Estimation of Total Flavonoids Content

The total flavonoids were extracted in methanol and estimated colorimetrically by using aluminum chloride based on the method described by Harborne [101]. The total flavonoid contents were calculated from a standard curve of quercetin and expressed as µg/g dry weight.

#### 4.4.5. Extraction and Estimation of Tannins Content

Tannins were extracted and estimated as described by Ejikeme et al. [102]. In a conical flask 1 g of powdered plant tissue was added to 100 mL of distilled water. Then, boiled gently for 1 h on an electric hot plate and then filtered. The diluted extract (10 mL) was added to a conical flask containing 50 mL of distilled water, 5.0 mL of Folin–Denis reagent, and 10 mL of saturated Na_2_CO_3_ solution for color development. The mixture was left to react in a water bath at 25 °C for 30 min. Optical density was read at 700 nm and the concentration was calculated from a standard curve of tannic acid as following:

(1) Tannic acid(mg/100 g)=C×extract volume×100/Aliquot volume×weight of sample

where *C* is the concentration of tannic acid read off the graph.

### 4.5. Biochemical Assay

#### 4.5.1. Extraction and Estimation of Total Soluble Proteins 

Total proteins were extracted by ground 0.5 g fresh tissue of leaves in 1 mL of phosphate buffer (0.1 M, pH 7.0) with a mortar and pestle and kept in ice. The protein concentration was estimated, and the absorbance was read at 595 nm on spectrophotometer based on the method described by Bradford [103].

#### 4.5.2. Determination of Protein Banding Pattern 

Total proteins were extracted from 0.5 g fresh tissue, the tissues were ground in liquid nitrogen. Then a few mL of tris buffer was added (1:2, tissue:buffer). The tris-HCl buffer contained 0.1 mM tris, pH 7.5, 4 mM B-mercaptoethanol, 0.1 mM EDTA-Na_2_, 10 mM KCl, and 10 mM MgCl_2_. The crude homogenate was centrifuged at 10,000× *g* for 20 min. The supernatant was used for gel analysis by SDS-polyacrylamide gel electrophoresis (SDS-PAGE) according to the method of Laemmli [104].

#### 4.5.3. Estimation of Proline 

Free proline was estimated by using ninhydrin reagent according to the method described by Bates et al. [105]. Proline concentration was measured from a standard curve of proline and expressed as µg/g fresh weight. 

#### 4.5.4. Extraction and Estimation of Malondialdehyde

Lipid peroxidation level was determined by measuring the amount of malondialdehyde (MDA) produced by the thiobarbituric acid reaction as described by Heath and Packer [106]. The crude extract was mixed with the same volume of a 0.5% thiobarbituric acid solution containing 20% trichloroacetic acid. The reaction mixture was incubated at 95 °C for 30 min and then cooled in an ice-bath. The mixture was centrifuged at 3000× *g* for 5 min. The absorbance of the supernatant was recorded at 532 and 600 nm. The MDA concentration was calculated by dividing the difference in absorbance (*A*_532_–*A*_600_) by its molar extinction coefficient (155 mM^−1^ cm^−1^), and the results expressed as μmol g^−1^ fresh weight.

### 4.6. Enzyme Extraction and Assays

#### Extraction and Assaying Activity of Certain Enzymes

The method adopted in enzyme extraction was that described by Mukheriee and Choudhuri, [107]. A fresh tissue (250 mg) was frozen in liquid nitrogen and finely grounded by pestle in a chilled mortar. The frozen powder was added to 10 mL of 100 mM phosphate buffer (KH_2_PO_4_/K_2_HPO_4_, pH 6.8). The homogenates were centrifuged at 20,000× *g* for 20 min. The supernatant was made up to a known volume with the same buffer and used as enzyme preparation for assaying the activity of certain enzymes.

Superoxide dismutase activity was measured according to the method of Dhindsa et al. [108]. A total of 3 mL of assay mixture contained 13 mM methionine, 0.025 mM *p*–nitro blue tetrazolium chloride (NBT), 0.1 mM EDTA, 50 mM phosphate buffer (pH 7.8), 50 mM sodium bicarbonate, and 0.5 mL enzyme extract. The reaction was started by adding riboflavin (0.002 mM) and incubating the tubes below two fluorescent lamps (15 W) for 15 min. The reaction was stopped by switching off the light and covering the tubes with black cloth. The tubes without enzyme developed maximal colors. A no irradiated complete reaction mixture served as blank. The absorbance was measured at 560 nm using Spekol spectrocolourimeter VEB Carl Zeiss. The enzyme activity was calculated as unit/mg protein.

Catalase activity was assayed according to the method of Hermans et al. [109]. The reaction mixture with final volume of 10 mL, containing 40 µL enzyme extract, was added to 9.96 mL of H_2_O_2_ contained in phosphate buffer, pH 7.0 (0.16 mL of 30% H_2_O_2_ to 100 mL of 50 mM phosphate buffer). CAT activity was determined by measuring the rate of change of H_2_O_2_ absorbance in 60 s using a Spekol spectrocolourimeter VEB Carl Zeiss at 250 nm. The blank sample was made by using buffer instead of enzyme extract. The enzyme activity was calculated as mM of H_2_O_2_/g FW/min.

Peroxidase activity was assayed according to the method of Kar and Mishra [110] after slight modifications. A total of 5 mL of the reaction mixture contained 300 μM of phosphate buffer (pH 6.8), 50 μM catechol, 50 μM H_2_O_2_ was added to 1 mL of crude enzyme extract. After incubation at 25 °C for 5 min, 1 mL of 10% H_2_SO_4_ was added for stopping the reaction. The optical density was measured at 340 nm, and the activity was expressed as the amount of quinone/g fresh weight/min.

APX activity was estimated following the method of Koricheva et al. [111] after slight modifications. The reaction mixture (10 mL) contained 5.5 mL of 50 mM phosphate buffer (pH 7.0), 0.5 mL of the enzyme extract, 1 mL 20 mM H_2_O_2_, 1 mL 20 mM EDTA, and 2 mL of 20 mM ascorbic acid. After ascorbate oxidation the rate of decrease in absorbance was recorded at 290 nm using a UV spectrophotometer (Unicam Heƛios Gamma and Delta). The enzyme activity was expressed as mM of ascorbate oxidized/g fresh weight/min.

### 4.7. Determination of Ascorbic Acid

Ascorbic acid was extracted estimated according to the methods of Kampfenkel et al. [112]. Fresh tissue (0.1 g) was homogenized in 1 mL 6% (*w/v*) trichloroacetic acid (TCA) solution and the homogenate was centrifuged at 12,000× *g* and 4 °C for 10 min. The supernatant was used for estimation of ascorbic acid.

### 4.8. Determination of Total Antioxidant Capacity

Total antioxidant capacity of the extract was evaluated by the phosphomolybdenum method as described by Prieto et al. [113]. A 0.3 mL extract was mixed with 3 mL of reagent solution (0.6 M sulfuric acid, 28 mM sodium phosphate, and 4 mM ammonium molybdate). The tubes containing the reaction mixture were incubated in water bath at 95 °C for 90 min. After cooling to room temperature, the absorbance of the mixture was measured at 695 nm against the blank. In the blank methanol (0.3 mL) was used in the place of extract. 

### 4.9. Statistical Analysis

Analyses of variance (ANOVA) for all experimental results presented in this study were calculated using SPSS v20.0 (SPSS Inc., Chicago, USA) analyzing software. Statistical significances of the means were compared with Duncan’s test at *p* ≤ 0.05 levels, the standard error (SE) of the means were presented in tables, and figures are means ± SE (*n* = 3).

## Figures and Tables

**Table 1:** Chemical analysis of soil samples collected from Saint Katherine protectorate (SKP) mountains at different altitudes (1600–1800, 1800–2000, and 2000–2200 m a.s.l.). Data are means of three replications ± SE.

Altitude (m a.s.l.)	pH Value	Anions (ppm)			Cations (ppm)			
		HCO_3_^−^	SO_4_^−^	Cl^−^	Ca^++^	Mg^++^	Na^+^	K^+^
1600–1800	8.2 ± 0.12 a	165.6 ± 7.5 b	95.6 ± 2 a	34 ± 4.9 a	79.5 ± 1.5 a	6.6 ± 0.4 c	28.0 ± 1.26 a	7 ± 1.15 b
1800–2000	7.8 ± 0.03 ab	228.7 ± 11.4a	59.1 ± 1.7 b	31.2 ± 5.1 a	42.2 ± 3.5 b	10.8 ± 0.37 a	8.6 ± 1.33 b	14.3 ± 0.88 b
2000–2200	7.6 ± 0.22 b	190.6 ± 5.6 b	18.6 ± 4.5 c	27.5 ± 1.3 a	74.0 ± 0.85 a	8.9 ± 0.29 b	8.0 ± 1.15 b	33.3 ± 3.84 a

Columns with different letters are significantly different at *p* < 0.05.

**Table 2:** Families, scientific names, altitude, and field photos of the studied endemic species.

Family	Plant Species	Altitude (m a.s.l.)			Field Photo
		(1600–1800)	(1800–2000)	(2000–2200)	
Lamiaceae	*Nepeta septemcrenata* Benth.	1630	1945	2038	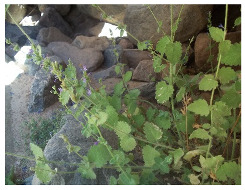
	*Origanum syriacum* subsp. *Sinaicum* (Boiss.) Greater and Burdet.	1630	1825	2038	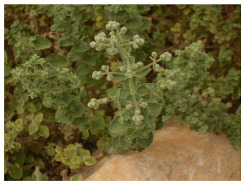
	*Phlomis aurea* Decene.	1710	1825	2038	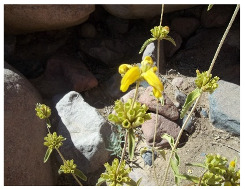
Rosaceae	*Rosa arabica* Crep.	1750	1940	2150	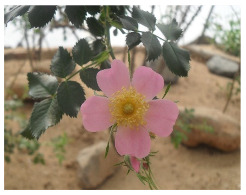
Caryophyllaceae	*Silene schimperiana* Boiss.	1750	1825	2110